# Age-dependent biphasic alterations and protein-activity decoupling of plasma BACE1 in major depressive disorder

**DOI:** 10.3389/fpsyt.2026.1917834

**Published:** 2026-08-26

**Authors:** Xiang Gao, Hang Li, Zuoli Sun, Min Liu, Yuhong Li, Weiwei Zhang, Jianping Chen, Rena Li

**Affiliations:** 1 Shanxi Bethune Hospital, Shanxi Academy of Medical Sciences, Third Hospital of Shanxi Medical University, Tongji Shanxi Hospital, Taiyuan, China; 2 Beijing Key Laboratory of Intelligent Drug Research and Development for Mental Disorders, National Clinical Research Center for Mental Disorders, National Center for Mental Disorders, Beijing Anding Hospital, Capital Medical University, Beijing, China; 3 Shanxi Provincial Innovation Center for Complex Formulation Technology of Anti-Tumor Drugs, Changzhi, China

**Keywords:** BACE1, biphasic alteration, depression-dementia continuum, executive function, gender dimorphism, major depressive disorder, protein-activity decoupling

## Abstract

**Background:**

Major depressive disorder (MDD) in late life is associated with an increased risk of Alzheimer's disease (AD), but the molecular basis for this depressiondementia continuum remains poorly understood. β-site amyloid precursor protein cleaving enzyme 1 (BACE1) processes both beneficial substrates (e.g., neuregulin-1 for synaptic plasticity) and pathological substrates (e.g., amyloid precursor protein for Aβ generation), making it a candidate molecular bridge. We hypothesized that plasma BACE1 levels would exhibit distinct age-dependent alteration patterns in MDD.

**Methods:**

Using a retrospective single-blind design, we enrolled 52 older (65–80 years) and 55 younger (30–40 years) MDD patients, with 75 age-matched healthy controls from the Beijing Biobank of Clinical Resources–Mental Disorders. Plasma BACE1 protein was measured by ELISA and enzymatic activity via FRET using an APP Swedish mutation substrate. Depression severity was assessed using HAMD; cognitive function using WCST.

**Results:**

Plasma BACE1 protein levels were significantly lower in MDD patients than in HCs (*P* = 0.029). A two-way ANOVA revealed a significant Age × Diagnosis interaction (*F*(1, 178) = 19.264, *P* < 0.001), formally supporting the biphasic pattern: young MDD patients showed a ~41% reduction (*P* < 0.001), while older MDD patients showed a numerical increase (*P* = 0.051). BACE1 enzymatic activity did not differ among groups, and no protein–activity correlation was observed within any subgroup, indicating a "decoupling" between protein expression and catalytic function. Plasma BACE1 protein levels in older MDD patients negatively correlated with executive dysfunction (WCST failure-to-maintain-set; *r* = −0.281, *P* = 0.044), while BACE1 activity in young MDD patients negatively correlated with age (*r* = −0.276, *P* = 0.042). A significant Age × Sex interaction (*F*(1, 178) = 6.756, P = 0.010) revealed an agedependent sexual dimorphism: young male controls had higher BACE1 levels than young female controls (*P* = 0.010), a pattern that reversed direction in older controls and was attenuated in both MDD subgroups.

**Conclusion:**

These findings reveal a substrate-selective, age-dependent, and sexinformed biphasic alteration of plasma BACE1 in MDD, supporting a unifying framework in which the same enzyme is associated with depressive pathology in young patients (via loss of beneficial substrate processing) and neurodegenerative transition in older patients (via pathological substrate cleavage), with the depression-dementia continuum as the mechanistic bridge.

## 1 Introduction

Major depressive disorder (MDD) is closely associated with Alzheimer’s disease (AD), collectively referred to as the “depression-dementia continuum” (1, 2). Late-onset depression (LOD) is often considered a prodromal phase or risk factor for AD, yet the molecular mechanisms underlying this transition remain largely elusive. Identifying shared biological markers between MDD and AD is crucial for understanding this continuum and for developing strategies to identify MDD patients at elevated risk for cognitive decline.

BACE1 (β-site amyloid precursor protein cleaving enzyme 1) is the rate-limiting enzyme initiating the formation of aggregation-prone Aβ1–42 in the brains of AD patients (3, 4). Beyond its well-known role in amyloidogenesis, BACE1 functions as a critical sheddase for multiple membrane proteins, including substrates relevant to synaptic plasticity and neuronal maintenance (5, 6). This multi-substrate property endows BACE1 with diverse physiological functions that extend far beyond amyloid pathology, raising the possibility that BACE1 dysregulation may contribute to neuropsychiatric conditions through multiple substrate-specific pathways.

Emerging evidence further suggests that BACE1 plays a dual role in psychiatric and cognitive functions. Recent phase 3 clinical trials of BACE1 inhibitors for AD were halted prematurely not only due to cognitive worsening but also because of severe neuropsychiatric adverse events, including worsening depression and suicidal ideation, indicating that the physiological basal activity of BACE1 is essential for mood homeostasis (7, 8). Furthermore, preclinical studies have confirmed that dysregulation of BACE1-mediated shedding of substrates like Nrg1 disrupts Nrg1-ErbB4 signaling, a pathway critically implicated in psychiatric homeostasis and mood regulation (5, 9). Genetic variations around the BACE1 cleavage site in APP can elevate or reduce BACE1’s APP-cleaving activity, exerting pathogenic or protective effects (3). These findings collectively underscore that BACE1 is not merely an amyloidogenic enzyme but a multifaceted regulator of synaptic and mood homeostasis, and that its dysregulation may have distinct functional consequences depending on the stage of life and the substrate milieu.

Despite this biological rationale, biomarker studies of BACE1 in MDD remain scarce. Multiple studies have found that both BACE1 activity and protein concentration in the blood are significantly increased in patients with mild cognitive impairment (MCI) or AD compared to healthy populations (10, 11). However, CSF studies of BACE1 in MDD are limited to two reports. One clinical study on cerebrospinal fluid (CSF) BACE1 in MDD reported that CSF BACE1 levels were significantly lower in MDD patients than in AD patients (12). More recently, Siafarikas et al. found that CSF BACE1 is elevated specifically in late-life depression patients with co-existing AD pathology, distinguishing them from depression patients without AD pathology (13). These studies highlight the complex, potentially stage-dependent role of BACE1 in the “depression-dementia continuum.” However, no study to date has systematically investigated plasma BACE1 protein levels and enzymatic activity in MDD patients across different age groups, leaving a critical gap in understanding the age-dependent trajectory of BACE1 dysregulation in depression.

In the present study, we aimed to investigate the alterations in plasma BACE1 protein levels and enzymatic activity in MDD patients of different ages and to explore their clinical significance. Plasma was chosen as the biofluid for its minimally invasive nature and suitability for repeated monitoring in clinical settings. By simultaneously measuring both BACE1 protein levels (by ELISA) and enzymatic activity (by FRET), we sought to capture potential dissociations between expression and function. Based on the dual role of BACE1 in synaptic homeostasis and amyloidogenesis, we hypothesized that plasma BACE1 levels would exhibit distinct age-dependent alteration patterns in MDD, potentially decreased in younger patients (reflecting loss of homeostatic BACE1) and increased in older patients (reflecting pathological upregulation reminiscent of early AD), thereby providing a molecular basis for the varying risks of cognitive decline between early- and late-onset depression.

## 2 Materials and methods

### 2.1 Study participants

Using a retrospective single-blind method, we selected 52 MDD patients aged 65–80 years, 55 MDD patients aged 30–40 years, 30 healthy controls aged 65–80 years, and 45 healthy controls aged 30–40 years from the Biobank of Beijing Anding Hospital. Inclusion criteria: Older adults aged 65–80 years; young adults aged 30–40 years; Han ethnicity; normal cognitive function; right-handed; no history of neurological trauma; no history of drug or substance abuse; full understanding of the study protocol and voluntary signature of informed consent. All subjects were adults, and consent from family members or guardians was also obtained. The diagnosis of MDD met the criteria of the Diagnostic and Statistical Manual of Mental Disorders, Fourth Edition (DSM-IV). DSM-IV-TR criteria were applied for diagnostic consistency with the original biobank enrollment period. Exclusion criteria: History of psychiatric disorders other than MDD; history of central or peripheral nervous system diseases; history of severe somatic diseases; family history of neurological disorders; pregnancy or lactation; history of food or drug allergies; history of blood draws or blood donation within 3 months prior to enrollment; inability to complete all study items; failure to sign informed consent. The diagnostic process was conducted by two experienced psychiatrists according to DSM-IV. The enrolled patients’ records were completed by trained raters, including personal information, disease status, past medical history, family history, age at onset and disease duration, treatment status, and scores on the Hamilton Depression Rating Scale HAMD, Hamilton Anxiety Rating Scale HAMA, Wisconsin Card Sorting Test WCST (14), Verbal Fluency Test (VFT) (15), Trail Making Test (TMT). The establishment of the Biobank was approved by the Ethics Committee of Beijing Anding Hospital [2014-68-201886XG-5], following a protocol approved by the hospital’s Human Research and Ethics Committee (16), in accordance with the latest version of the Declaration of Helsinki. All subjects voluntarily signed informed consent. Plasma samples and clinical data were retrieved for analysis.

### 2.2 Plasma protein and enzymatic activity assays

Total plasma protein was quantified using the Pierce BCA Protein Assay Kit (Thermo Fisher Scientific). Plasma BACE1 protein levels were measured using an ELISA kit (EHBACE1, Thermo fisher, USA). Based on the cleavage sites of APP (Swedish mutation) reported in the literature, we synthesized the Fluorescence Resonance Energy Transfer (FRET) fluorogenic substrates, (Abz)-SEVNLDAEFRK-(Dnp)-NH_2_. Briefly, the FRET substrate stock was diluted to its working concentration (100 μM) in sodium acetate buffer (50 mM HOAc, 100 mM NaCl, pH 4.0), and a 50 μL aliquot was mixed with 50 μL of plasma in a light-protected black 96-well plate. The fluorescence intensity (excitation: 320 nm; emission: 420 nm) was continuously monitored at 37 °C for 30–60 minutes using a microplate reader. The maximum reaction velocity (Vmax) was determined from the initial 5–10 minutes of the kinetic curve, and BACE1 activity was ultimately normalized to the total plasma protein concentration (BACE1 activity = Vmax/Total plasma protein).

### 2.3 Statistical analysis

All experimental data were entered into a database and analyzed using SPSS Statistics 26.0, with graphs generated using GraphPad Prism 8. Pearson correlation analysis was used to evaluate the correlations between plasma BACE1 levels/activity and other clinical indicators. The significance level was set at *α* = 0.05. Normality was assessed using the Shapiro-Wilk test. Continuous variables following a normal distribution were expressed as mean ± standard deviation (SD), and comparisons between two groups were performed using independent samples t-tests. Normality was assessed using the Shapiro-Wilk test for each subgroup. For comparisons in which either subgroup deviated significantly from normality (*P* < 0.05), we additionally performed the non-parametric Mann-Whitney U test as confirmation. All *P* values reported are two-tailed.

## 3 Results

### 3.1 Decreased plasma BACE1 protein levels in MDD patients

**Figure 1:**
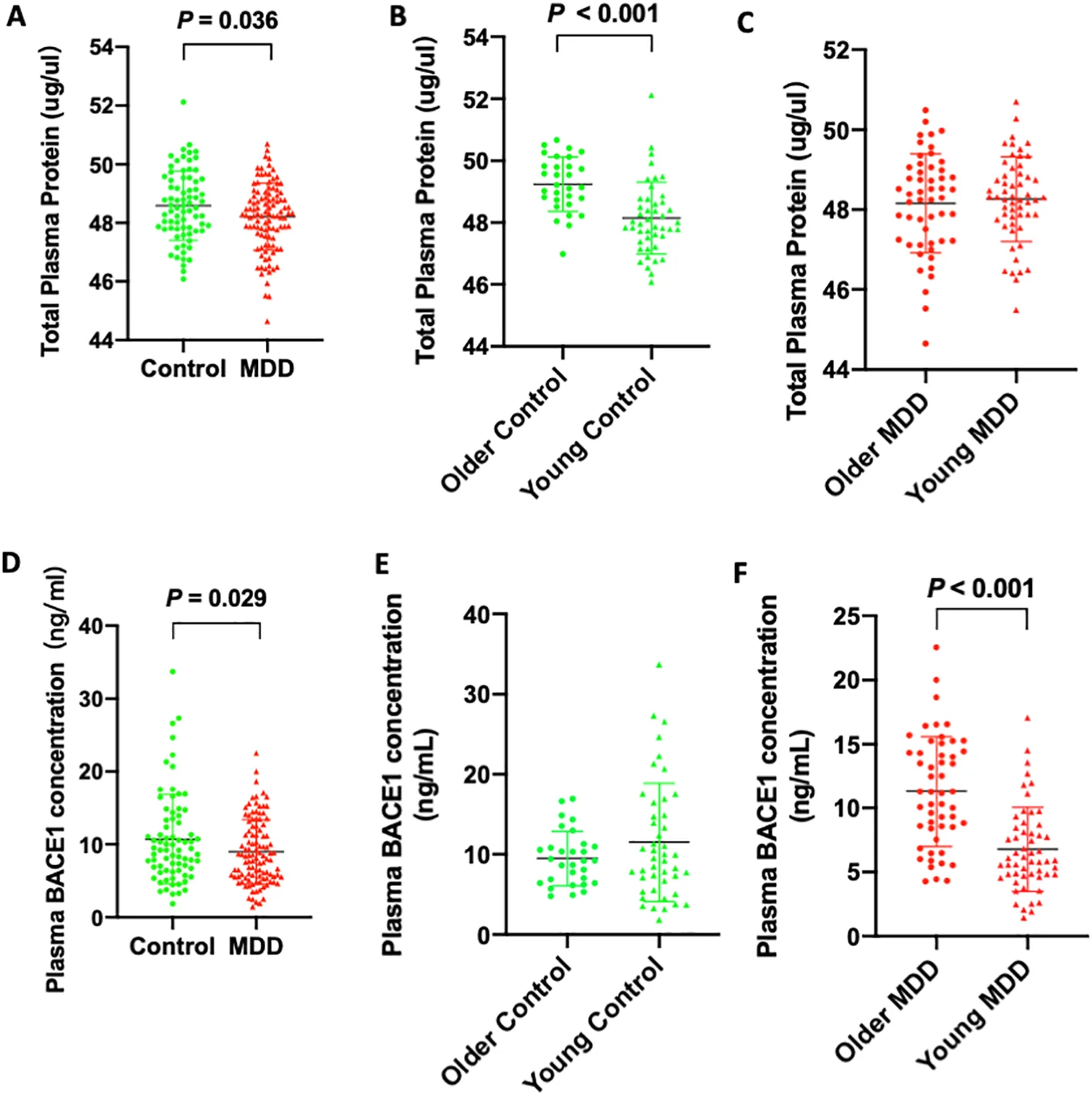
Comparison of total plasma protein and plasma BACE1 protein concentrations between MDD patients and healthy controls, with age-stratified subgroup analysis. **(A)** Total plasma protein levels in the overall MDD and control groups. **(B)** Total plasma protein levels in older versus young controls. **(C)** Total plasma protein levels in older versus young MDD patients. **(D)** Plasma BACE1 protein concentrations in the overall MDD and control groups. **(E)** Plasma BACE1 protein concentrations in older versus young controls. **(F)** Plasma BACE1 protein concentrations in older versus young MDD patients. Data are presented as mean ± SD. *P* values are shown above each group comparison. Young adults: 30–40 years; older adults: 65–80 years.

In the overall comparison, both total plasma protein concentration (*P* = 0.036, **Figure 1A**) and plasma BACE1 protein concentration (*P* = 0.029, **Figure 1D**) were significantly lower in MDD patients than in healthy controls. There were no significant differences between the MDD and control groups regarding mean age, age distribution, gender distribution, or plasma BACE1-APP activity (*P* > 0.05, **Table 1**). Age-stratified analysis of total plasma protein revealed that older controls had significantly higher total plasma protein levels than young controls (*P* < 0.001, **Figure 1B**), whereas no significant difference was observed between older and young MDD patients (*P* > 0.05, **Figure 1C**), suggesting that MDD may attenuate the age-related increase in total plasma protein observed in healthy individuals. Notably, plasma BACE1 protein concentrations exhibited a divergent age-dependent pattern: while BACE1 levels did not differ significantly between older and young controls (*P* > 0.05, **Figure 1E**), older MDD patients had significantly higher BACE1 levels than young MDD patients (*P* < 0.001, **Figure 1F**).

**Table 1:** Comparison between the control and MDD groups.

Variable	Control (n = 75)	MDD (n = 107)	*χ2/t*	*P*
Age (years)	46.60 (14.71)	50.64 (17.15)	-1.700	0.091
Female (%)	40 (53.3)	67 (62.6)	1.569	0.210
Young subgroup (%)	45 (60.0)	55 (51.4)	1.317	0.251
Total plasma protein (μg/μL)	48.585 (1.19)	48.213 (1.15)	**2.119**	**0.036**
Plasma BACE1 concentration (ng/mL)	10.693 (6.15)	8.972 (4.42)	**2.198**	**0.029**
Plasma BACE1-APP activity (Vmax/μg)	12.152 (4.06)	12.281 (4.26)	-0.205	0.838

Bold values indicate statistically significant differences (P < 0.05).

To formally test the biphasic pattern, we conducted a two-way between-subjects ANOVA with Age group (young vs. older) and Diagnosis (MDD vs. control) as fixed factors on plasma BACE1 protein concentration. The main effect of Diagnosis fell short of significance, *F* (1, 178) = 3.820, *P* = 0.052, consistent with the biphasic pattern — the opposite-direction changes in young and older MDD patients partially cancel in the pooled sample. Critically, the Age × Diagnosis interaction was highly significant, *F* (1, 178) = 19.264, *P* < 0.001, partial *η*² = 0.098 (large effect), formally supporting the biphasic pattern: young MDD patients showed lower BACE1 and older MDD patients showed higher BACE1 relative to age-matched controls. The main effect of Age was not significant, *F* (1, 178) = 2.798, *P* = 0.096. The overall model was significant, *F* (3, 178) = 10.463, *P* < 0.001, *R²* = 0.150, adjusted *R²* = 0.136.

### 3.2 Upward trend of plasma BACE1 protein in older MDD patients

**Figure 2:**
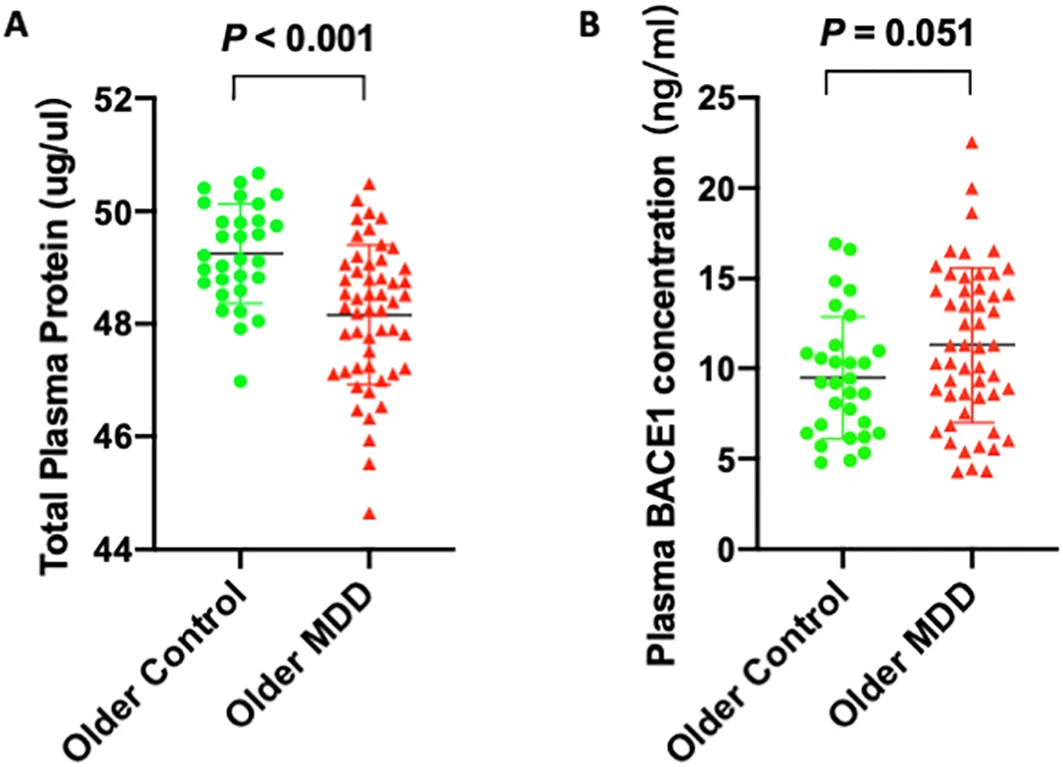
Alterations of total plasma protein and plasma BACE1 protein concentrations in the older population. **(A)** Total plasma protein levels in older healthy controls (n = 30) versus older MDD patients (n = 52). **(B)** Plasma BACE1 protein concentrations in older healthy controls versus older MDD patients. Data are presented as mean ± SD. *P* values are shown above each group comparison.

In the older population, total plasma protein levels were significantly lower in MDD patients than in healthy controls (*P* < 0.001, **Figure 2A**), consistent with the overall trend observed in the total cohort. In contrast, plasma BACE1 protein concentrations in older MDD patients exhibited an upward trend compared with older controls (*P* = 0.051, **Figure 2B**), a direction opposite to the overall decrease seen in the total MDD cohort (**Figure 1D**). There was no significant difference in plasma BACE1-APP activity between the two groups (*P* = 0.868, **Table 2**). Although the age of older MDD patients was significantly greater than that of older controls (*P* < 0.001, **Table 2**), age had no significant correlation with either total plasma protein or BACE1 concentration within this subgroup (*P* > 0.05 for both), suggesting that the observed BACE1 alteration is disease-related rather than age-driven.

**Table 2:** Baseline characteristics of the older population.

Variable	Older control (n = 30)	Older MDD (n = 52)	*χ2/t*	*P*
Female (%)	18 (60.0)	33 (63.5)	0.097	0.756
Age (years)	64.00 (3.12)	67.62 (5.32)	**-3.881**	**< 0.001**
Total plasma protein (μg/μL)	49.249 (0.88)	48.160 (1.24)	**4.227**	**< 0.001**
Plasma BACE1 concentration (ng/mL)	9.479 (3.39)	11.293 (4.30)	**-1.982**	**0.051**
Plasma BACE1-APP activity (Vmax/μg)	12.815 (3.61)	12.662 (4.19)	0.167	0.868

Continuous variables compared using Student’s two-tailed independent samples t-test (or Mann-Whitney U test for non-normally distributed data, as indicated by Shapiro-Wilk test). Categorical variables compared using Pearson’s chi-square test. P < 0.05 was considered statistically significant. Effect sizes: Cohen’s d for t-tests (small=0.20, medium=0.50, large=0.80). The mean age of 64.00 +/- 3.12 years in the Older Control group reflects two participants who were 64 years and 7–11 months at enrollment, meeting the >=65 years criterion at the time of data extraction. Bold values indicate statistically significant differences (P < 0.05).

To address potential confounding by the age imbalance between older MDD patients (67.62 ± 5.32 years) and older controls (64.00 ± 3.12 years), we performed an ANCOVA with chronological age as covariate. After age adjustment, the group difference remained significant, *F* (1, 79) = 7.035, *P* = 0.010, partial *η*² = 0.082; age covariate: *F* = 4.571, *P* = 0.036. Estimated marginal means at the mean age of the older subsample (66.29 years) were 11.559 ng/mL (*SE* = 0.556, 95% *CI*: 10.453-12.665) for older MDD patients and 9.018 ng/mL (*SE* = 0.745, 95% *CI*: 7.535-10.501) for older controls, a difference of 2.541 ng/mL (95% *CI*: 0.634-4.447). This indicates that the elevation observed in older MDD patients is not attributable to the age difference between groups alone.

### 3.3 Clinical significance of plasma BACE1 protein levels and activity in older MDD

To explore the clinical relevance of plasma BACE1 alterations in older MDD patients, Pearson correlation analyses were performed between BACE1 protein concentration or enzymatic activity and a comprehensive panel of clinical indicators, including age, disease duration, age at onset, HAMD and HAMA scores, and multiple WCST sub-indices (**Table 3**). Plasma BACE1 protein levels were significantly and negatively correlated with the “failure to maintain set” index of the WCST (*r* = −0.281, *P* = 0.044), indicating that higher BACE1 levels are associated with better executive function maintenance. No significant correlations were observed between BACE1 protein levels and other clinical indicators (all *P* > 0.05). Plasma BACE1-APP enzymatic activity showed no significant correlations with any clinical indicator in the older MDD group (all *P* > 0.05, **Table 3**), further supporting the “decoupling” between BACE1 protein expression and its APP-cleaving function.

**Table 3:** Correlations between plasma BACE1 protein/activity and clinical indicators in older MDD patients.

Variable	Plasma BACE1 concentration		Plasma BACE1-APP activity	
	*r*	*P*	*r*	*P*
Age (years)	-0.215	0.126	-0.090	0.524
Disease duration (months)	-0.042	0.768	-0.080	0.575
Age at onset (years)	-0.214	0.128	-0.055	0.700
HAMD score	0.135	0.341	-0.141	0.319
HAMA score	-0.137	0.334	-0.013	0.926
WCST				
-Total responses	-0.205	0.145	-0.038	0.787
-Categories completed	0.100	0.482	0.023	0.872
-Correct responses	0.007	0.962	0.093	0.510
-Correct response rate (%)	0.031	0.828	0.077	0.589
-Incorrect responses	-0.097	0.496	-0.105	0.459
-First category responses completed	-0.112	0.428	-0.262	0.061
-Conceptual level responses (%)	-0.097	0.495	-0.223	0.111
-Perseverative responses	0.064	0.652	0.215	0.126
-Perseverative errors	-0.084	0.553	-0.234	0.096
-Perseverative error percentage (%)	0.085	0.548	0.104	0.461
-Non-perseverative errors	0.063	0.657	-0.003	0.985
-Failure to maintain set	**-0.281**	**0.044**	-0.072	0.610

Continuous variables compared using Student’s two-tailed independent samples t-test (or Mann-Whitney U test for non-normally distributed data, as indicated by Shapiro-Wilk test). Categorical variables compared using Pearson’s chi-square test. P < 0.05 was considered statistically significant. Effect sizes: Cohen’s d for t-tests (small=0.20, medium=0.50, large=0.80). Bold values indicate statistically significant differences (P < 0.05).

### 3.4 Significantly decreased plasma BACE1 protein levels in young MDD patients

**Figure 3:**
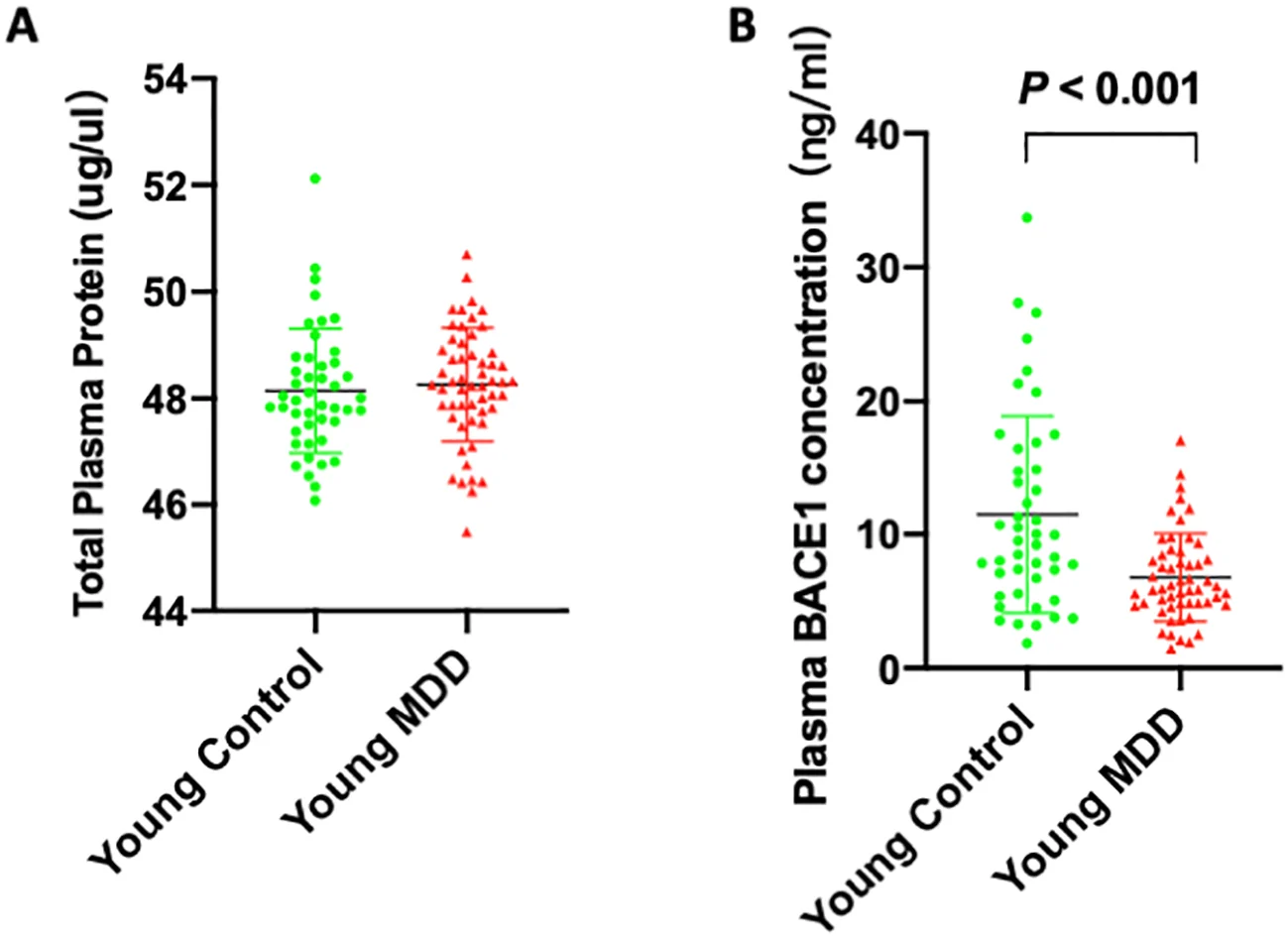
Alterations of total plasma protein and plasma BACE1 protein concentrations in the young population. **(A)** Total plasma protein levels in young healthy controls (n = 45) versus young MDD patients (n = 55). **(B)** Plasma BACE1 protein concentrations in young healthy controls versus young MDD patients. Data are presented as mean ± SD. *P* values are shown above each group comparison.

In the young population, there were no significant differences between MDD patients and healthy controls in age (*P* = 0.549), gender ratio (61.8% vs. 48.9% female, *P* = 0.195), or total plasma protein concentration (*P* = 0.590, **Figure 3A**; **Table 4**). In striking contrast, plasma BACE1 protein concentrations in young MDD patients were significantly lower than those in young healthy controls (*P* < 0.001, **Figure 3B**), representing an approximately 41% reduction. This marked downregulation is the primary driver of the overall BACE1 decrease observed in the total MDD cohort (**Figure 1D**). Plasma BACE1-APP activity did not differ significantly between the two groups (*P* = 0.809, **Table 4**), again demonstrating a decoupling between BACE1 protein expression and enzymatic activity.

**Table 4:** Baseline characteristics of the young population.

Variable	Young control (n = 45)	Young MDD (n = 55)	*χ2/t*	*P*
Female (%)	22 (48.9)	34 (61.8)	1.679	0.195
Age (years)	35.00 (3.65)	34.58 (3.29)	0.602	0.549
Total plasma protein (μg/μL)	48.14 (1.17)	48.26 (1.06)	-0.540	0.590
Plasma BACE1 concentration (ng/mL)	11.502 (7.37)	**6.777 (3.28)**	**3.987**	**< 0.001**
Plasma BACE1-APP activity (Vmax/μg)	11.710 (4.31)	11.920 (4.33)	-0.242	0.809

BACE1 protein levels in both young subgroups deviated from normality (Shapiro-Wilk: young control *W* = 0.904, *P* = 0.001; young MDD *W* = 0.941, *P* = 0.010). The young MDD-vs-control comparison was therefore additionally confirmed using the Mann-Whitney U test (*U* = 741.000, *Z* = -3.440, *P* = 0.001, consistent with the t-test result). Bold values indicate statistically significant differences (P < 0.05).

### 3.5 Negative correlation between plasma BACE1-APP activity and age in young MDD

In young MDD patients, plasma BACE1 protein concentrations showed no significant correlations with any clinical or cognitive indicator, including age, age at onset, vital signs, anthropometric measures, HAMD scores, VFT sub-scores, or TMT performance (all *P* > 0.05, **Table 5**). However, plasma BACE1-APP enzymatic activity was significantly and negatively correlated with age (*r* = −0.276, *P* = 0.042, **Table 5**), suggesting that even within the narrow 30–40 year age window, younger patients tend to exhibit higher BACE1 enzymatic activity. No other significant correlations were observed between BACE1 activity and the remaining clinical indicators (all *P* > 0.05). The absence of a corresponding correlation between BACE1 protein levels and age further underscores the dissociation between protein expression and enzymatic activity in this population.

**Table 5:** Correlations between plasma BACE1 protein/activity and clinical indicators in young MDD patients.

Variable	Plasma BACE1 concentration		Plasma BACE1-APP activity	
	*r*	*P*	*r*	*P*
Age (years)	-0.009	0.947	**-0.276**	**0.042**
Age at onset (years)	0.168	0.245	0.132	0.359
Heart rate (beats/min)	0.138	0.324	0.032	0.818
Systolic blood pressure (mmHg)	0.137	0.322	-0.133	0.337
Diastolic blood pressure (mmHg)	-0.116	0.404	-0.170	0.219
Height (cm)	0.139	0.312	0.006	0.964
Weight (kg)	0.074	0.592	-0.011	0.938
BMI	0.013	0.926	-0.008	0.953
Hip circumference (cm)	0.188	0.177	-0.100	0.475
Waist circumference (cm)	0.123	0.371	-0.042	0.762
HAMD score	0.165	0.229	-0.048	0.729
VFT test				
-T1 (Fruits)	-0.050	0.722	-0.068	0.626
-T2 (Vegetables)	-0.052	0.706	-0.095	0.496
-T3 (Animals)	0.084	0.547	0.108	0.437
-T4 (Cities)	-0.004	0.978	-0.070	0.614
TMT test				
-A completion time	0.123	0.385	0.231	0.099
-A error count	-0.046	0.756	0.053	0.715
-B completion time	0.126	0.379	0.173	0.225
-B error count	0.013	0.933	0.008	0.956

Continuous variables compared using Student’s two-tailed independent samples t-test (or Mann-Whitney U test for non-normally distributed data, as indicated by Shapiro-Wilk test). Categorical variables compared using Pearson’s chi-square test. P < 0.05 was considered statistically significant. Effect sizes: Cohen’s d for t-tests (small=0.20, medium=0.50, large=0.80). Bold values indicate statistically significant differences (P < 0.05).

### 3.6 Decoupling between BACE1 protein levels and enzymatic activity across all groups

**Figure 4:**
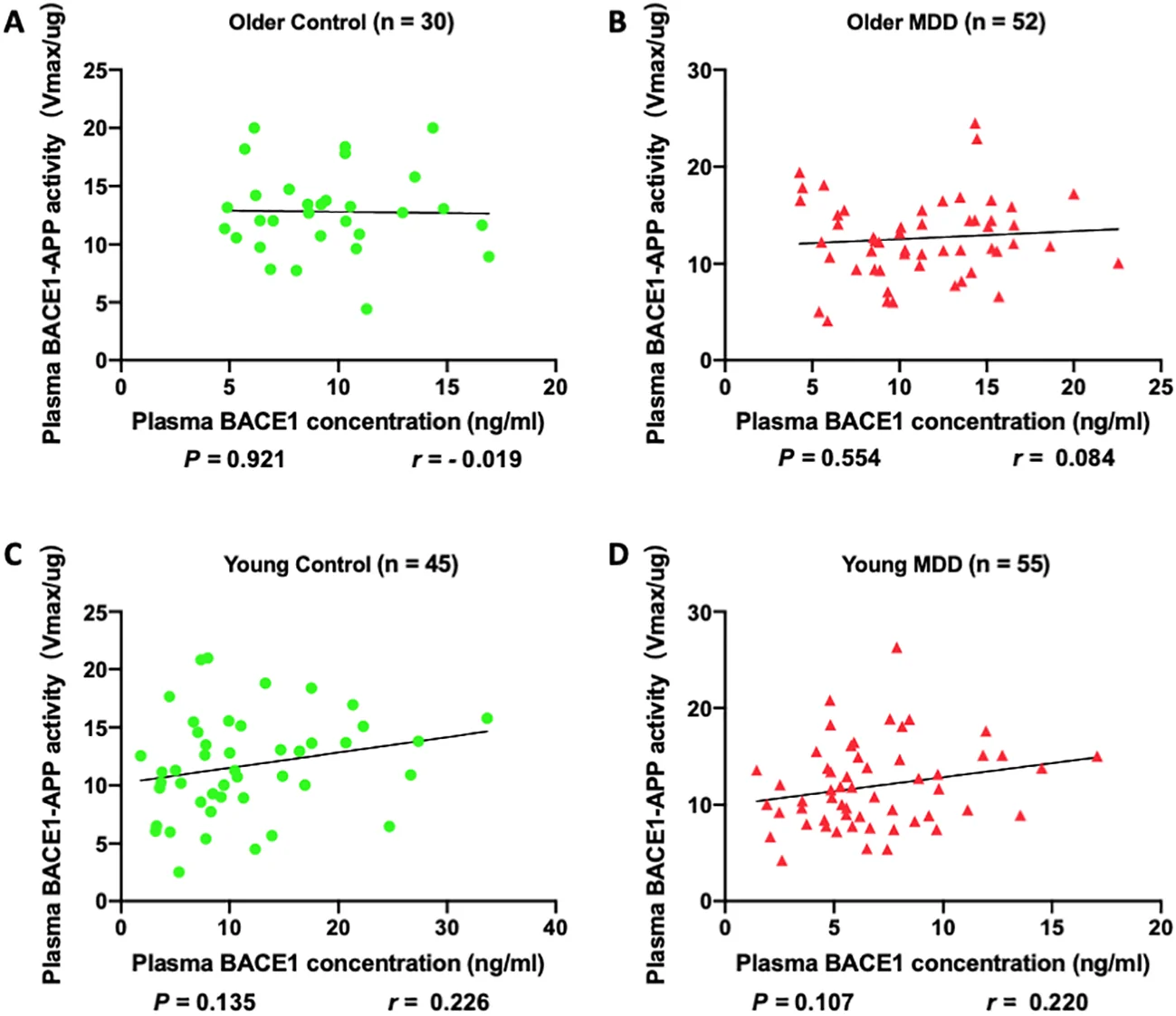
Correlation between plasma BACE1 protein concentrations and BACE1-APP enzymatic activities across four subgroups. **(A)** Older controls (n = 30). **(B)** Older MDD patients (n = 52). **(C)** Young controls (n = 45). **(D)** Young MDD patients (n = 55). Pearson correlation coefficients (r) and *P* values are shown in each panel. Dashed lines represent linear regression fits. ns, not significant. Young adults: 30–40 years; older adults: 65–80 years.

To directly assess the relationship between BACE1 protein expression and its APP-cleaving activity, Pearson correlation analyses were performed within each of the four subgroups (**Figure 4**). In the older control group, no significant correlation was observed between plasma BACE1 protein concentration and BACE1-APP enzymatic activity (*r* = −0.019, *P* = 0.921, **Figure 4A**). Similarly, in the older MDD group, the correlation was not significant (*r* = 0.084, *P* = 0.554, **Figure 4B**). In the young control group, a weak positive trend was noted but did not reach statistical significance (*r* = 0.226, *P* = 0.135, **Figure 4C**). A comparable weak positive trend was observed in the young MDD group (*r* = 0.220, *P* = 0.107, **Figure 4D**). The absence of a significant protein–activity correlation in any subgroup, regardless of age or disease status, confirms a decoupling between BACE1 protein expression and its APP-cleaving function. Methodological constraints of the FRET-based activity assay are discussed in Section 4.6.

### 3.7 Age-dependent gender dimorphism of plasma BACE1 protein concentration

**Figure 5:**
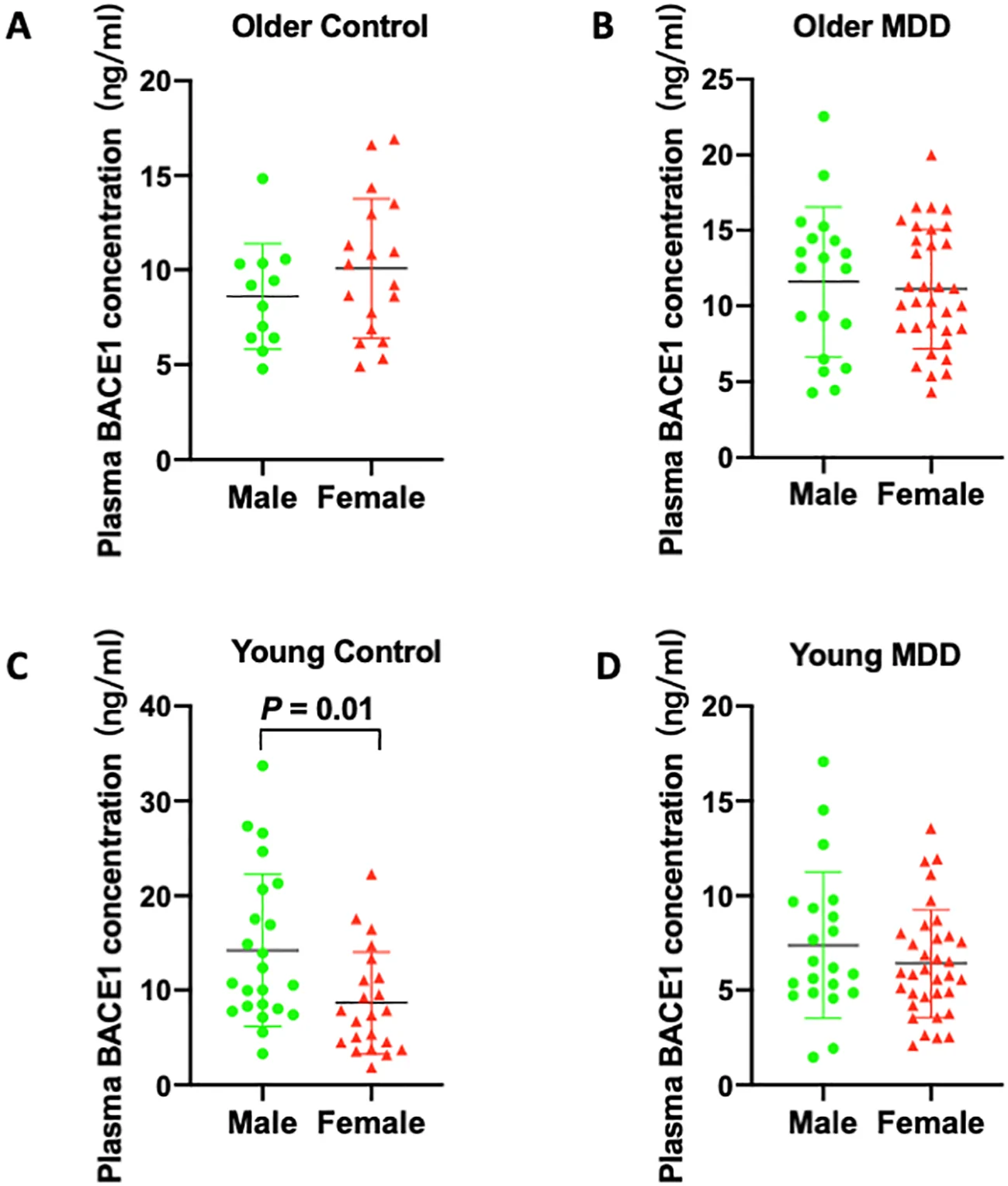
Gender-stratified analysis of plasma BACE1 protein concentrations across age and diagnosis groups. Scatter-box plots showing plasma BACE1 protein concentrations (ng/mL) in male (green circles) and female (red triangles) participants within each of the four study groups: **(A)** Older Control (Male: n = 12, Female: n = 18), **(B)** Older MDD (Male: n = 19, Female: n = 33), **(C)** Young Control (Male: n = 23, Female: n = 22), and **(D)** Young MDD (Male: n = 21, Female: n = 34). Box plots display the median (central line), interquartile range (box), and data range (whiskers). Individual data points are overlaid to show distribution density. P values were derived from independent samples t-tests comparing Male vs. Female within each group. Young adults: 30–40 years; older adults: 65–80 years.

Given the notably high standard deviation of plasma BACE1 protein concentration observed in the young control group (**Table 1**; **Figure 1D**), a *post-hoc* gender-stratified analysis was performed to examine whether gender-specific effects contribute to the observed variance. All four groups were stratified by sex, and the descriptive statistics are presented in **Table 6**. In healthy controls, plasma BACE1 protein concentration exhibited a significant gender dimorphism that was age-dependent. In the young control group, male participants had markedly higher BACE1 levels than female participants (14.22 ± 8.07 vs. 8.67 ± 5.40 ng/mL, *t* = 2.699, *P* = 0.010, **Figure 5C**), representing an approximately 64% elevation. In contrast, no significant gender difference was observed in the older control group (8.59 ± 2.78 vs. 10.08 ± 3.69 ng/mL, *t* = - 1.189, *P* = 0.244, **Figure 5A**), where the direction of the difference was reversed (female > male) and the magnitude was substantially smaller. This age-dependent loss of BACE1 gender dimorphism in healthy aging suggests a potential interaction between sex-related factors and BACE1 regulation. Notably, the pronounced gender difference in young controls accounts for the unusually high standard deviation observed in the merged young control group (11.50 ± 7.37 ng/mL, **Table 1**). The pooling of high-BACE1 males with low-BACE1 females inflated the within-group variance, underscoring the importance of considering gender as a biological variable in BACE1 biomarker studies. In both MDD groups, the gender dimorphism of BACE1 was attenuated (**Figures 5B, D**). This pattern suggests that MDD may disrupt the physiological gender-specific regulation of BACE1, consistent with the disease-related BACE1 alterations described in the preceding sections. No significant gender differences were observed for age, total plasma protein concentration, or BACE1-APP enzymatic activity in any of the four groups (all *P* > 0.05), indicating that the gender effect was specific to BACE1 protein concentration rather than a general plasma protein alteration or a uniform shift in enzymatic activity.

**Table 6:** Gender-stratified analysis of plasma BACE1 protein concentration, enzymatic activity, and clinical characteristics across age and diagnosis groups.

Variable	Older control		Older MDD		Young control		Young MDD	
	Male (n = 12)	Female (n = 18)	Male (n = 19)	Female (n = 33)	Male (n = 23)	Female (n = 22)	Male (n = 21)	Female (n = 34)
Age (years)	63.92 (2.47)	64.06 (3.56)	66.90 (5.26)	68.03 (5.39)	34.96 (3.84)	35.05 (3.53)	34.91 (3.72)	34.66 (3.24)
Total plasma protein (μg/μL)	49.22 (0.75)	49.27 (0.99)	47.80 (1.26)	48.37 (1.20)	48.23 (1.45)	48.05 (0.79)	48.08 (1.28)	48.31 (0.96)
Plasma BACE1 concentration (ng/mL)	8.59 (2.78)	10.08 (3.69)	11.59 (4.96)	11.12 (3.94)	14.22 (8.07)	**8.67** **(5.40)** **	7.38 (3.88)	6.41 (2.86)
Plasma BACE1-APP activity (Vmax/μg)	12.70 (3.08)	12.90 (4.01)	13.00 (5.06)	12.46 (3.66)	12.18 (4.56)	11.22 (4.09)	12.01 (3.78)	11.68 (4.70)
Cohen’s d (95% CI)	-0.44		0.11		0.81		0.30	

Data are presented as mean (SD). Gender comparisons (Male vs. Female) within each group were performed using independent samples t-tests. **P = 0.01 for the Male vs. Female comparison within the Young Control group. Continuous variables compared using Student’s two-tailed independent samples t-test (or Mann-Whitney U test for non-normally distributed data, as indicated by Shapiro-Wilk test). Categorical variables compared using Pearson’s chi-square test. P < 0.05 was considered statistically significant. Effect sizes: Cohen’s d for t-tests (small=0.20, medium=0.50, large=0.80). Bold values indicate statistically significant differences (P < 0.05).

To formally test the sexually dimorphic pattern, we performed a two-way ANOVA with Age group (young vs. older) and Sex (male vs. female) as fixed factors on plasma BACE1 protein concentration. The Age group × Sex interaction was significant, *F* (1, 178) = 6.756, *P* = 0.010, partial *η*² = 0.037), as was the main effect of Sex, *F* (1, 178) = 4.717, *P* = 0.031, partial *η*² = 0.026); the main effect of Age group fell short of significance, *F* (1, 178) = 3.652, *P* = 0.058, partial *η*² = 0.020). The overall model was significant, *F* (3, 178) = 6.104, *P* = 0.001, *R*² = 0.093, adjusted *R*² = 0.078). The pattern of the interaction was consistent with the sexually dimorphic interpretation: the age-related difference in BACE1 was substantially larger in females (older 10.75 ± 3.85 vs. young 7.29 ± 4.16, Δ = +3.46 ng/mL) than in males (older 10.43 ± 4.45 vs. young 10.95 ± 7.23, Δ = -0.52 ng/mL). In young healthy controls, males exhibited significantly higher BACE1 levels than females (14.22 +/- 8.07 vs. 8.67 +/- 5.40 ng/mL, *P* = 0.010, *Cohen’s d* = 0.81, large effect), whereas in older controls, females showed nominally higher levels (*Cohen’s d* = -0.44, medium effect; *P* = 0.244, not significant).

## 4 Discussion

The present study reveals for the first time an age-dependent biphasic alteration of plasma BACE1 protein levels in MDD patients and its correlation with executive function. Our findings demonstrate that while plasma BACE1 protein levels are generally lower in MDD patients compared to healthy controls, this overall decrease is driven by the young MDD subgroup. Conversely, older MDD patients exhibited an upward trend in plasma BACE1 protein levels. Furthermore, we identified a “decoupling” phenomenon between BACE1 protein expression and its APP-cleaving activity, and a specific correlation between BACE1 levels and executive dysfunction in older patients.

### 4.1 The age-dependent biphasic alteration: a substrate selectivity framework

The most striking finding is the age-dependent biphasic alteration of plasma BACE1 protein levels in young versus older MDD patients, formally supported by a significant Age × Diagnosis interaction in two-way between-subjects ANOVA, *F* (1, 178) = 19.264, *P* < 0.001). Notably, the main effect of Diagnosis did not reach the conventional significance threshold, *F* (1, 178) = 3.820, *P* = 0.052, which is itself consistent with the biphasic pattern: the opposite-direction changes in young (decrease) and older (increase) MDD patients partially cancel each other in the pooled sample, diluting the overall disease effect. The main effect of Age was also non-significant, *F* (1, 178) = 2.798, *P* = 0.096. In young MDD patients, BACE1 protein was significantly reduced by approximately 41%. In contrast, older MDD patients showed an upward trend in BACE1protein, aligning with studies of late-life depression (LOD) where CSF BACE1 is elevated, particularly in the presence of amyloid pathology (13).

The mechanistic basis for this biphasic pattern can be understood through the lens of BACE1’s substrate selectivity. BACE1 is not a promiscuous protease; it processes a diverse array of membrane proteins with markedly different affinities, kinetics, and subcellular compartmentalization (6, 17). Critically, these substrates fall into two functional categories: “beneficial” substrates whose cleavage supports physiological neuronal function—including Nrg1 for myelination and synaptic plasticity (5, 9, 18, 19), CHL1 for axon guidance and synaptic maintenance (20), and the voltage-gated sodium channel β-subunit (Navβ) for neuronal excitability (6, 17); and “pathological” substrates whose excessive cleavage is associated with neurodegeneration-including APP for Aβ production (3, 4) and α-Klotho, whose BACE1-mediated cleavage at the FT site depletes this neuroprotective protein (21). This substrate dichotomy predicts that the net functional consequence of BACE1 dysregulation depends critically on which substrate class is preferentially affected—a determination that is, in turn, governed by the patient’s age, the local substrate milieu, and the subcellular trafficking of BACE1.

In the young brain, where amyloid pathology is minimal and synaptic remodeling is active, the dominant BACE1 substrates are likely the “beneficial” category-particularly Nrg1 and CHL1, which are essential for ongoing myelination and circuit refinement (18–20). When BACE1 protein is significantly reduced, as observed in our young MDD patients (~41% decrease), the processing of these high-turnover beneficial substrates would be preferentially compromised. Indeed, BACE1 heterozygous knockout mice, which retain ~50% of BACE1 protein, exhibit significant deficits in Nrg1 signaling and myelination despite having no amyloid pathology (18, 19, 22), demonstrating that beneficial substrate processing is exquisitely sensitive to BACE1 dosage even when overall residual activity appears adequate. Consistent with this model, recent quantitative synthetic MRI evidence has confirmed abnormal myelin integrity as a prominent feature in MDD patients (23), providing a structural correlate for the functional deficit predicted by our substrate selectivity framework. The resulting hypomyelination-particularly in prefrontal-limbic circuits-would disrupt the neural connectivity essential for mood regulation, providing a plausible mechanistic link between reduced BACE1 and the depressive phenotype in young patients.

Conversely, in the aging brain, the substrate milieu shifts. Age-associated increases in APP expression and neuroinflammatory signaling may elevate the relative availability of “pathological” substrates, rendering BACE1 upregulation increasingly deleterious. In older MDD patients, the observed upward trend in BACE1 protein may reflect a compensatory response-initially driven by homeostatic feedback to counteract synaptic decline-but the consequence is increased cleavage of APP and α-Klotho. We have demonstrated that BACE1 specifically cleaves α-Klotho at the FT site (21), and that plasma α-Klotho is reduced in elderly MDD patients (16), suggesting that the “BACE1-α-Klotho axis” shifts from physiological maintenance to pathological degeneration in late-life depression. This substrate milieu shift provides a mechanistic explanation for why the same enzyme (BACE1) can be associated with depression in the young (through loss of beneficial substrate processing) and with neurodegeneration in the old (through gain of pathological substrate processing).

### 4.2 The decoupling phenomenon: substrate competition and differential affinity

A notable observation in our study is the dissociation between BACE1 protein levels and enzymatic activity. In young MDD patients, despite a 41% reduction in BACE1 protein, APP-based FRET enzymatic activity was unchanged, while older MDD patients showed both unchanged protein and activity. This “decoupling” suggests that BACE1 protein levels alone do not directly determine its APP-cleaving activity in plasma. This dissociation has been noted in AD, where BACE1 activity increases with cognitive decline even when protein expression remains unaltered (24).

Several factors may contribute to this decoupling. First, post-translational modifications-including glycosylation, acetylation, and palmitoylation-can modulate BACE1 catalytic efficiency independently of total protein levels (25–27). Altered glycosylation patterns in MDD patients could shift BACE1’s specific activity without changing its abundance.

Second, and more fundamentally, substrate competition and differential affinity provide a compelling explanation for the decoupling. BACE1 substrates do not share equal access to the enzyme. APP, the substrate with the highest known catalytic efficiency for BACE1, may continue to be efficiently processed even when total BACE1 protein is substantially reduced-as a high-affinity substrate, APP would outcompete lower-affinity substrates (such as Nrg1 or CHL1) for the remaining enzyme (17). This “substrate competition” model predicts that moderate reductions in BACE1 protein would preferentially impair the processing of low-affinity beneficial substrates while leaving high-affinity APP cleavage largely intact-precisely the pattern observed in our young MDD patients, who showed a 41% reduction in BACE1 protein but no change in APP-based FRET activity.

Conversely, in older MDD patients with an upward trend in BACE1 protein, the excess enzyme may be increasingly directed toward APP and α-Klotho cleavage-substrates whose availability is elevated in the aging brain-without proportionally increasing the net APP-cleaving activity measured by FRET, if the additional BACE1 is sequestered in different subcellular compartments (e.g., endosomal vs. cell-surface pools) or exists in a different glycosylation state with altered substrate preference (25–27). The FRET assay, by utilizing a soluble Swedish-mutation APP substrate, measures the catalytic capacity of all BACE1 molecules toward APP in a cell-free system, and may therefore not reflect the substrate-selective changes occurring *in vivo* where compartmentalization and co-localization are critical determinants of substrate access.

This substrate-competition framework has profound implications for interpreting BACE1 biomarker studies. It suggests that measuring only APP-cleaving activity-as most clinical BACE1 assays do, including ours-systematically misses alterations in the processing of other substrates that may be more relevant to psychiatric pathology. The decoupling we observed is therefore a biological pattern that cannot be attributed solely to assay artifact, although the precise mechanism remains to be established. The apparent shift in BACE1’s substrate preference in MDD cannot be confirmed without BACE1-inhibitor control experiments and broader substrate panels. This interpretation is consistent with the recent recognition that BACE1 inhibitors failed in AD clinical trials partly because they blocked processing of beneficial substrates (Nrg1, CHL1) indiscriminately along with APP (7, 8, 20), producing the very neuropsychiatric adverse events that halted their development.

### 4.3 Correlation with executive function: the dual-substrate trade-off

In older MDD patients, we found that higher BACE1 protein levels were associated with better executive function, as measured by the WCST “failure to maintain set” index (*r* = -0.281, *P* = 0.044), indicating that patients with higher BACE1 had fewer set-maintenance failures and thus better executive performance. No such correlation was found in young MDD patients.

This seemingly paradoxical finding can be reconciled by considering the substrate selectivity framework outlined above. In older MDD patients, the “upward trend” of BACE1 simultaneously affects two categories of substrates with opposing functional consequences. On one hand, higher BACE1 levels may sustain the processing of beneficial substrates—particularly Nrg1-ErbB4 signaling, which is essential for prefrontal myelin maintenance and synaptic plasticity (5, 9, 23). The WCST “failure to maintain set” index specifically reflects prefrontal executive function, a domain critically dependent on intact frontostriatal myelination. Patients who can mount a higher BACE1 response may therefore preserve Nrg1-mediated myelination in prefrontal circuits, sustaining better executive performance-explaining the observed negative correlation between BACE1 levels and set-maintenance failures.

On the other hand, the same elevated BACE1 may inexorably accelerate the cleavage of pathological substrates-APP, increasing Aβ production, and α-Klotho, depleting the neuroprotective protein we have shown to be reduced in elderly MDD (16, 21). This creates a temporal trade-off: in the short term, upregulated BACE1 preserves cognitive function by maintaining beneficial substrate processing; in the long term, the same elevation is associated with amyloid pathology and α-Klotho depletion, accelerating the transition toward AD. The “double-edged sword” is thus not abstract-it is grounded in the concrete duality of BACE1’s substrate spectrum, with the same enzyme concurrently executing beneficial and pathological cleavages.

This model carries a critical translational implication. The failure of BACE1 inhibitor trials in AD (7, 8) can be reinterpreted through this lens: by indiscriminately blocking all BACE1-mediated cleavage, these inhibitors not only reduced Aβ production but also disrupted the processing of Nrg1, CHL1, and other beneficial substrates (6, 20)-producing cognitive worsening and neuropsychiatric adverse events that mirrored the very symptoms they sought to prevent. Our finding that higher BACE1 correlates with better executive function in older MDD provides clinical evidence supporting this interpretation. Future therapeutic strategies targeting BACE1 may need to achieve substrate-selective modulation-preserving beneficial substrate processing while selectively inhibiting pathological APP and α-Klotho cleavage-a concept that remains aspirational but is increasingly motivated by the substrate-selectivity framework.

### 4.4 BACE1 activity decline with age in young MDD: early loss of compensatory buffer

An unexpected finding was the negative correlation between BACE1 enzymatic activity and age within the young MDD group (*r* = -0.276, *P* = 0.042), observed within the narrow 30–40 year window. This suggests that even within this relatively young cohort, patients closer to age 30 tend to exhibit higher BACE1 activity, while those closer to 40 show progressively lower activity. Importantly, this correlation was specific to enzymatic activity, not protein levels, which were uniformly reduced across the young MDD group regardless of age.

One interpretation is that BACE1 activity serves as a homeostatic buffer in early-life depression. In the earliest stages of MDD (potentially closer to onset), compensatory mechanisms may upregulate BACE1 catalytic efficiency (through post-translational modifications such as enhanced glycosylation or altered subcellular trafficking) to maintain beneficial substrate processing despite reduced total protein. As the disease progresses chronically (reflected in older age within this cohort), this compensatory capacity may become exhausted, leading to a decline in BACE1 activity. This “compensatory exhaustion” model is consistent with the broader concept of allostatic load, wherein prolonged physiological adaptation ultimately fails. If confirmed in longitudinal studies, this finding would suggest that the window for therapeutic intervention to preserve BACE1’s homeostatic function may be narrow-potentially early in the disease course before compensatory mechanisms are depleted.

### 4.5 Age-dependent gender dimorphism of plasma BACE1: the estrogen–ERE–BACE1 axis

The gender-stratified analysis (**Table 6**) revealed a striking and previously unreported finding: the direction of the gender dimorphism in plasma BACE1 protein concentration is age-dependent. In young healthy controls, males exhibited significantly higher BACE1 levels than females (14.22 ± 8.07 vs. 8.67 ± 5.40 ng/mL, *P* = 0.010), whereas in older controls, this pattern was reversed—females showed nominally higher levels than males (10.08 ± 3.69 vs. 8.59 ± 2.78 ng/mL)—although the difference did not reach statistical significance (*P* = 0.244), likely due to the limited sample size (n = 30). This directional inversion across age strata is now formally supported by a significant Age group × Sex interaction in two-way ANOVA (*F* (1, 178) = 6.756, *P* = 0.010), although the MDD-specific three-way Age × Sex × Diagnosis test remains a target for future work with larger samples.

This age-dependent reversal is remarkably consistent with the known regulatory axis of estrogen on BACE1 transcription. Pioneering work by Li and colleagues established that brain endogenous estrogen levels determine the responsiveness of the BACE1 system to hormonal modulation: in female Alzheimer’s transgenic mice, brain estrogen depletion upregulated BACE1 expression and impaired neprilysin (NEP)-mediated Aβ clearance, while estrogen replacement restored both parameters—but only when endogenous brain estrogen was sufficiently depleted (28). This work was extended by the comprehensive review by Li et al. (29), which synthesized evidence that brain estrogen is not merely a systemic hormone acting centrally, but a locally synthesized neuromodulator whose age- and sex-dependent decline fundamentally reshapes cognitive trajectories and AD vulnerability. Building on this framework, Cui et al. (30) recently demonstrated that estrogen represses BACE1 gene transcription through a functional estrogen response element (ERE) in the BACE1 promoter, providing a direct molecular mechanism for the female-specific suppression of BACE1 expression. In premenopausal women, sustained high levels of 17β-estradiol activate ERα-mediated transcriptional repression of BACE1, maintaining lower BACE1 protein levels relative to males. Following menopause, the precipitous decline in estrogen relieves this transcriptional suppression, allowing BACE1 expression to rise in older females—consistent with both our observed directional inversion and the established epidemiology of AD, where postmenopausal women bear a disproportionate risk (29, 30).

In males, the age-related trajectory of BACE1 is governed by a different hormonal regulator. Testosterone has been shown to reduce BACE1 activity, mRNA, and protein expression in the brain independently of its aromatization to estrogen (31). Unlike the sharp estrogen decline at menopause, age-related testosterone decline in males is gradual (~1–2% per year), resulting in a slow upward drift in BACE1 that is modest compared to the postmenopausal female surge. This asymmetric hormonal architecture—sudden estrogen loss in women versus gradual testosterone decline in men—provides a parsimonious explanation for the cross-over pattern: young males have higher BACE1 than young females (no estrogen suppression in males; strong ERE-mediated suppression in females), while older females overtake older males (derepressed BACE1 transcription post-menopause).

Importantly, this gender dimorphism is consistent with the findings of Vergallo et al. (32), who reported that plasma BACE1 concentrations were significantly higher in women than in men among cognitively normal older individuals at risk for AD, further supporting the postmenopausal reversal. Our study extends this observation by revealing the opposite pattern in young controls and, crucially, by demonstrating that MDD attenuates the gender dimorphism: in both older MDD and young MDD, the male–female difference in BACE1 was substantially reduced compared to the corresponding control groups. This disease-associated “de-gendering” suggests that MDD may disrupt the physiological hormonal regulation of BACE1—potentially through hypothalamic-pituitary-gonadal (HPG) axis dysfunction, which is well-documented in MDD—or that MDD-specific pathological processes (e.g., neuroinflammation, cortisol dysregulation) override the modulatory effects of sex hormones on BACE1 transcription.

The gender dimorphism observed here was specific to BACE1 protein concentration; no significant gender differences were found for BACE1-APP enzymatic activity, total plasma protein, or age in any group (all *P* > 0.05). This specificity mirrors the decoupling phenomenon described in Section 4.2 and reinforces the interpretation that BACE1 protein levels and catalytic activity are regulated by distinct mechanisms—in this case, transcriptional control by sex hormones affects total protein abundance without proportionally altering the catalytic capacity measured by the FRET assay.

From a methodological standpoint, this finding has immediate implications for BACE1 biomarker research. The markedly elevated variance in the young control group (SD = 7.374, compared to 2.78–4.96 in other groups) is directly attributable to the mixing of high-BACE1 males and low-BACE1 females. Future studies measuring plasma BACE1, particularly in young populations, should routinely report gender-stratified data or include sex as a covariate. Failure to do so introduces systematic variance that can obscure group differences and inflate Type II error—a methodological concern that may partly explain the heterogeneous BACE1 findings across published MDD and AD studies.

The sexually dimorphic interpretation is hypothesis-generating and requires direct testing in future studies with sex hormone measurements and a three-way Age × Sex × Diagnosis design. If confirmed, this framework predicts that premenopausal women’s low BACE1 baseline represents a reduced homeostatic reserve of beneficial substrate processing, potentially rendering them more vulnerable to the BACE1-lowering effect of MDD - a hypothesis that could be directly tested in prospective cohorts with hormonal profiling.

### 4.6 Limitations and conclusion

Several limitations should be acknowledged. First, the cross-sectional design precludes causal inferences, and the multiple comparisons across age and disease strata require that subgroup t-tests and correlations be interpreted as exploratory rather than confirmatory. The two-way ANOVA formally testing the biphasic pattern (Age × Diagnosis interaction: *F (1*, 178) = 19.264, *P* < 0.001) provides the strongest statistical support and does not require correction for multiple comparisons; however, replication in independent cohorts is needed, and longitudinal studies are required to determine whether BACE1 alterations precede or follow depression onset.

Second, plasma BACE1 may not fully reflect central nervous system BACE1 activity, although growing evidence supports a correlation between peripheral and central BACE1 levels (10, 11). Additionally, while the sample size was sufficient to detect the primary effects, it may be underpowered for subtle interaction effects—particularly the three-way (age × diagnosis × sex) interactions identified in the gender-stratified analysis.

Third, the FRET-based BACE1 activity assay has four methodological constraints: (i) the APP Swedish mutation substrate reflects only BACE1’s APP-cleaving activity, potentially missing alterations in the processing of other substrates (e.g., Nrg1, CHL1, α-Klotho) more relevant to MDD pathology; (ii) activity was normalized to total plasma protein rather than BACE1 protein level specifically, which could introduce confounding when total protein differs between groups; (iii) plasma levels of BACE1 substrates were not measured concurrently; and (iv) plasma contains multiple proteases with overlapping substrate specificity, and BACE1-inhibitor negative control reactions (e.g., parallel reactions with β-secretase inhibitor IV) could not be performed as the biobank plasma samples have been depleted. The decoupling between BACE1 protein and APP-cleaving activity therefore cannot be definitively attributed to BACE1-specific catalytic changes versus altered contributions from other proteases. Future studies should develop multiplexed substrate-specific assays, employ BACE1 protein-normalized activity, and include inhibitor controls to directly test the substrate competition model.

Fourth, the age imbalance between older MDD patients and older controls (67.62 vs. 64.00 years) was statistically significant; however, ANCOVA confirmed that the BACE1 group difference remained significant after age adjustment, *F* (1, 79) = 7.035, *P* = 0.010, partial *η*² = 0.082). The age covariate itself was significant, *F* = 4.571, *P* = 0.036), indicating that age contributes to BACE1 variance but does not account for the disease-related difference.

Fifth, the gender-stratified analysis was *post-hoc* and exploratory, and plasma sex hormone levels (estradiol, testosterone, FSH) were not measured. The Age_group × Sex interaction (F(1, 178) = 6.756, P = 0.010, partial η² = 0.037) was formally tested on the full sample (MDD + control combined); a definitive test of sexual dimorphism specifically within MDD patients would require a three-way Age × Sex × Diagnosis design with larger sample sizes. The estrogen-BACE1 regulatory mechanism proposed in Section 4.5 is inferred from literature evidence (28–30) rather than directly validated. Independent replication with hormonal profiling is needed.

Sixth, medication status was listed among the collected biobank variables but could not be reliably retrieved for this analysis. As BACE1 is sensitive to inflammation and oxidative stress, and effective antidepressant treatment tends to reduce inflammatory markers, medication use remains an uncontrolled confounder that could partly influence the observed BACE1 differences, particularly in the young MDD group. Future studies with systematic medication documentation are needed to assess this possibility directly.

Seventh, the absence of a significant protein-activity correlation within any subgroup should not be over-interpreted as proof of true biological decoupling. The subgroup sample sizes (n = 30-55) provide limited power to detect moderate correlations, and the positive trends observed in the young subgroups (*r* = 0.226 and 0.220) are consistent with an incipient coupling the study was not powered to confirm. We therefore distinguish between absence of evidence and evidence of absence.

In conclusion, this study demonstrates an age-dependent biphasic alteration of plasma BACE1 protein in MDD—significantly reduced in young patients but elevated in older patients—formally supported by a significant Age × Diagnosis interaction, *F* (1, 178) = 19.264, *P* < 0.001. The substrate selectivity-threshold framework integrates this biphasic pattern with BACE1’s dual beneficial (Nrg1, CHL1, Navβ) and pathological (APP, α-Klotho) substrate roles, providing a mechanistic bridge between the mood and neurodegeneration arms of BACE1 biology. The gender dimension—formally supported by a significant Age_group × Sex interaction, *F* (1, 178) = 6.756, *P* = 0.010, adds a third axis of heterogeneity that future studies must address. Together, these findings position plasma BACE1 as a promising but age-, sex-, and disease-stratified biomarker candidate for MDD, with direct implications for the design of BACE1-targeted therapeutic strategies.

## Data Availability

The raw data supporting the conclusions of this article will be made available by the authors, without undue reservation.
